# Patterning and gastrulation defects caused by the *t^w18^* lethal are due to loss of *Ppp2r1a*

**DOI:** 10.1242/bio.023200

**Published:** 2017-06-15

**Authors:** Lisette Lange, Matthias Marks, Jinhua Liu, Lars Wittler, Hermann Bauer, Sandra Piehl, Gabriele Bläß, Bernd Timmermann, Bernhard G. Herrmann

**Affiliations:** 1 Max Planck Institute for Molecular Genetics, Department Developmental Genetics, Ihnestraße 63-73, Berlin 14195, Germany; 2 Free University Berlin, Department of Biology, Chemistry and Pharmacy, Takustrasse 3, Berlin 14195, Germany; 3 Max Planck Institute for Molecular Genetics, Sequencing Core Facility, Ihnestraße 63-73, Berlin 14195, Germany; 4 Charité-University Medicine Berlin, Institute for Medical Genetics, Campus Benjamin Franklin, Hindenburgdamm 30, Berlin 12203, Germany

**Keywords:** *t* haplotype, *t^w18^*, Mesoderm, Primitive streak, Signal transduction, PP2A phosphatase

## Abstract

The mouse *t* haplotype, a variant 20 cM genomic region on Chromosome 17, harbors 16 embryonic control genes identified by recessive lethal mutations isolated from wild mouse populations. Due to technical constraints so far only one of these, the *t^w5^* lethal, has been cloned and molecularly characterized. Here we report the molecular isolation of the *t^w18^* lethal. Embryos carrying the *t^w18^* lethal die from major gastrulation defects commencing with primitive streak formation at E6.5. We have used transcriptome and marker gene analyses to describe the molecular etiology of the *t^w18^* phenotype. We show that both WNT and Nodal signal transduction are impaired in the mutant epiblast, causing embryonic patterning defects and failure of primitive streak and mesoderm formation. By using a candidate gene approach, gene knockout by homologous recombination and genetic rescue, we have identified the gene causing the *t^w18^* phenotype as *Ppp2r1a*, encoding the PP2A scaffolding subunit PR65alpha. Our work highlights the importance of phosphatase 2A in embryonic patterning, primitive streak formation, gastrulation, and mesoderm formation downstream of WNT and Nodal signaling.

## Abstract (precis)

**Summary:** The *t^w18^* lethal, a ‘classical’ mutation, affects gastrulation and embryo patterning. This article shows that phosphatase PP2A is dysfunctional in *t^w18^* and essential for Nodal and WNT signaling in the epiblast.

## INTRODUCTION

Between 6-25% of individuals in wild house mouse populations carry a *t* haplotype, a variant form of the *t* complex spanning over 20 cM (∼40 Mbp) of Chromosome 17 (Chr 17) (Carroll et al., 2004; reviewed in Silver, 1985; Sugimoto, 2014). This region comprises many important developmental regulators, among them *Pou5f1* (*Oct4*), *Nkx2-5*, *Axin* and *Brachyury* (*T*), as well as 16 recessive lethal mutations, known as *t* lethals, which are encoded by different *t* haplotypes and affect early embryonic development (Klein et al., 1984; Bennett, 1975; Sugimoto, 2014). Due to particularities of the *t* haplotypes, to date only one *t* lethal, *t^w5^*, has been identified and characterized at the molecular level (Sugimoto et al., 2012). The *t* lethal *t^w18^* and related *t* lethals of the same complementation group (*t^4^*, *t^9^*) were described about 50 years ago (Bennett and Dunn, 1960; Moser and Gluecksohn-Waelsch, 1967). They cause strong gastrulation defects with striking overgrowth of the primitive streak (PS) and bulging of cells into the amniotic cavity, commencing on the seventh and prominent on the eighth to ninth day of gestation, followed by embryonic death one day later. In contrast, normal development requires the ingression of epiblast cells at the PS, epithelial-to-mesenchymal transition (EMT) and migration of single mesodermal cells in between the epiblast and the visceral endoderm (VE) (reviewed in Arnold and Robertson, 2009). Thus, the failure of mesoderm formation characterizes the embryonic phenotype of these mutants. However, the molecular cause and consequences have remained obscure.

The *t^w18^* haplotype originated from a rare recombination event between a *t* haplotype and the wild-type chromosome across a large inversion, causing a large deletion of at least 3.3 Mbp comprising many genes (Búcan et al., 1987; Barclay et al., 1996). Here we have determined the extent of the deletion and, by using a candidate gene approach, have identified *Ppp2r1a* as the gene causing the *t^w18^* mutant phenotype. It encodes the scaffolding subunit Aα (PR65alpha) of protein phosphatase 2A (PP2A). The PP2A family of serine-threonine phosphatases is implicated in many cellular processes, such as the regulation of signaling pathways, cell cycle, DNA replication and apoptosis (Virshup, 2000; Janssens and Goris, 2001), and its deregulation is associated with a range of human diseases like Alzheimer's disease and different types of cancer (Eichhorn et al., 2009; Vafai and Stock, 2002). We describe the identification of the *t^w18^* lethal and provide a detailed molecular characterization of the *t^w18^* gastrulation defects.

## RESULTS

### Nodal and WNT signal transduction are impaired in homozygous *t^w18^* embryos

**Fig. 1.**
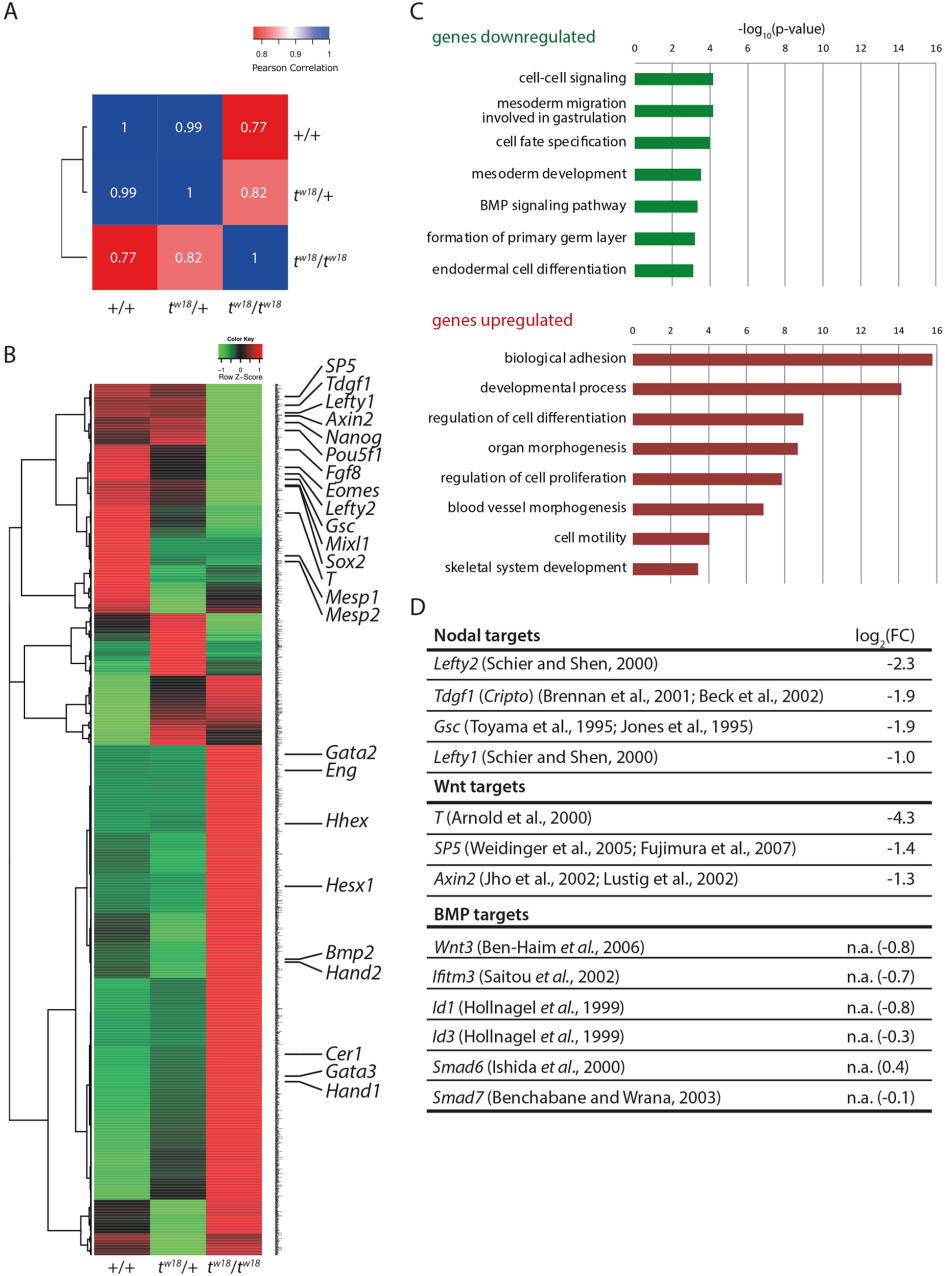
**Transcriptome analysis reveals downregulation of WNT and Nodal targets in *t^w18^* mutant embryos.** (A) Pearson correlation coefficient of next generation sequencing data from wild-type, homozygous and heterozygous *t^w18^* embryos. (B) Heatmap of 2539 genes differentially expressed genes in wild-type, *t^w18^*/+ and *t^w18^*/*t^w18^* embryos with log_2_(FC)≥1. (C) GO term enrichment analyses of genes dysregulated between *t^w18^*/*t^w18^* and wild-type log_2_(FC≥1). GO terms were analyzed for downregulated (upper panel) and upregulated (lower panel) genes. The diagrams shows selected GO terms with corresponding −Log_10_ of the *P*-value that were given by the Ontologizer tool. A background of genes expressed in the dataset with FPKM 2 was used. (D) Examples of Nodal and WNT targets downregulated in *t^w18^* homozygotes; BMP targets were not affected (n.a.).

Prior to this study, *t^w18^*/*t^w18^* mutant embryos have not been characterized at the molecular level. We started the molecular characterization of *t^w18^*/*t^w18^* mutants by analyzing the transcriptome of homozygous, heterozygous and wild-type embryos at embryonic day (E)6.5, when malformations start to develop, by RNA-sequencing (RNA-seq). Embryos were dissected in two parts: the embryo proper consisting of epiblast and surrounding visceral endoderm (VE), which was used for RNA extraction, and the extra-embryonic tissue, required for genotyping. The Pearson correlation coefficient (PCC) of the sequencing data showed a high similarity between wild-type and heterozygous *t^w18^* samples (PCC 0.99, Fig. 1A). The *t^w18^*/*t^w18^* sample differed from both the wild-type (PCC 0.77) and the heterozygous sample (PCC 0.82). After removing genes with FPKM (fragments per kilobase of exon per million fragments mapped) values <1 in the three samples, log_2_ of the fold change (FC)≥1.0 for any comparable pair was used as the threshold to define the affected genes, and we got 2539 deferentially expressed genes (Fig. 1B).

Among the most strongly downregulated genes in *t^w18^/t^w18^* mutants is the pan-mesodermal marker *Brachyury* [*T*, log_2_(FC)=−4.3], indicating that mesoderm formation is severely compromised (Wilkinson et al., 1990; Arnold et al., 2000; Kubo et al., 2004). Other downregulated mesoderm control genes comprise *Eomesodermin* (*Eomes*, Russ et al., 2000), *Mesp1* (Saga et al., 1996), *Mesp2* (Saga et al., 1997), *Mixl1* (Pearce and Evans, 1999), and the signal molecule *Fgf8* (Crossley and Martin, 1995). A gene ontology (GO) term analysis revealed an enrichment of terms related to cellular signaling, mesoderm formation and cell fate specification among the downregulated genes, while biological adhesion was the most significant term related to upregulated genes (Fig. 1C). These data are consistent with the severe gastrulation defects observed previously in homozygous *t^w18^* embryos (Bennett and Dunn, 1960).

Since the GO analysis pointed to impaired signaling, we searched the list of dysregulated genes for factors involved in signaling pathways. We found that targets of Nodal signaling in the epiblast, including *Tdgf1* (*Cripto*, Brennan et al., 2001; Beck et al., 2002), *Gsc* (Toyama et al., 1995; Jones et al., 1995), and *Lefty2* (Schier and Shen, 2000), are downregulated in *t^w18^*/*t^w18^* embryos (Fig. 1D). Expression of *Nodal* itself, the Nodal convertases *Furin* (*Spc1*) and *Pcsk6* (*Spc4*, Beck et al., 2002), and *Smad2/3* (Kumar et al., 2001), as well as *Smad4* (Lagna et al., 1996), the intracellular transmitters of Nodal signaling, are not affected (Table S1). However, *Cer1* (Piccolo et al., 1999), *Hesx1* (Thomas et al., 1995), and *Hhex* (Keng et al., 1998), markers of the anterior visceral endoderm (AVE) are upregulated, indicating that an anterior pole might be established. The fact that the latter markers are also dependent on Nodal signaling suggests that Nodal signaling is not generally impaired, but primarily in the epiblast.

Similarly, the WNT target genes *T* (Arnold et al., 2000), *Sp5* (Weidinger et al., 2005), and *Axin2* (Jho et al., 2002; Lustig et al., 2002) are downregulated, indicating that WNT signaling in the epiblast is also affected in the mutant. Expression of the *Wnt3* morphogen itself and the final effector β-catenin, encoded by *Ctnnb1*, are not significantly dysregulated (Table S1).

Since Nodal and WNT signaling are required for pattern formation, formation of the PS, and mesoderm induction, the molecular data suggested that all these essential embryonic processes are impaired in *t^w18^*/*t^w18^* embryos (Schier and Shen, 2000).

In contrast to WNT and Nodal signaling, dysregulation of bone morphogenetic protein (BMP) target genes, such as *Wnt3* (Ben-Haim et al., 2006), *Ifitm3* (*Fragilis*, Saitou et al., 2002), *Id1/Id3* (Hollnagel et al., 1999), *Smad6* (Ishida et al., 2000), or *Smad7* (Benchabane and Wrana, 2003) was not apparent in the transcriptome data from mutant embryos (Fig. 1D). Moreover, *Bmp2* is more strongly expressed in the mutant (Fig. 1B). BMP signaling is the major inducer of extra-embryonic and lateral mesoderm (Tonegawa et al., 1997; Arnold et al., 2006). Among the most highly upregulated mesodermal genes is the TGFβ co-receptor *Endoglin* (*Eng*), which is involved in extra-embryonic mesoderm formation and vasculogenesis (Arthur et al., 2000; Jonker and Arthur, 2001). Among the lateral mesoderm regulators several Gata factors (*Gata2*, *Gata3*), *Hand1* and *Hand2* are more highly expressed in the mutant transcriptome. These data indicate that mesoderm formation downstream of BMP signaling appears unaffected in the mutant.

We also observed that the pluripotency genes *Nanog*, *Oct4* and *Sox2*, which are expressed in the epiblast (Nichols et al., 1998; Avilion et al., 2003; Mitsui et al., 2003), are expressed at a lower level in the mutants.

In summary, the transcriptome data indicated that the *t^w18^* mutants are characterized by impaired Nodal and WNT signaling downstream of the ligands.

### Homozygous *t^w18^* embryos are disorganized

Transcriptome data per se do not provide spatial resolution, and a reduction or increase of marker gene expression at the transcriptome level does not allow us to distinguish whether the expression per cell or the relative number of cells expressing the marker is altered. Thus, to characterize the etiology of the *t^w18^* phenotype in embryos we performed whole-mount *in situ* hybridizations (WISHs) of marker genes on E6.5, E7.5, E8.5, and E9.5 embryos derived from inter-crosses of heterozygous *t^w18^* animals.

**Fig. 2.**
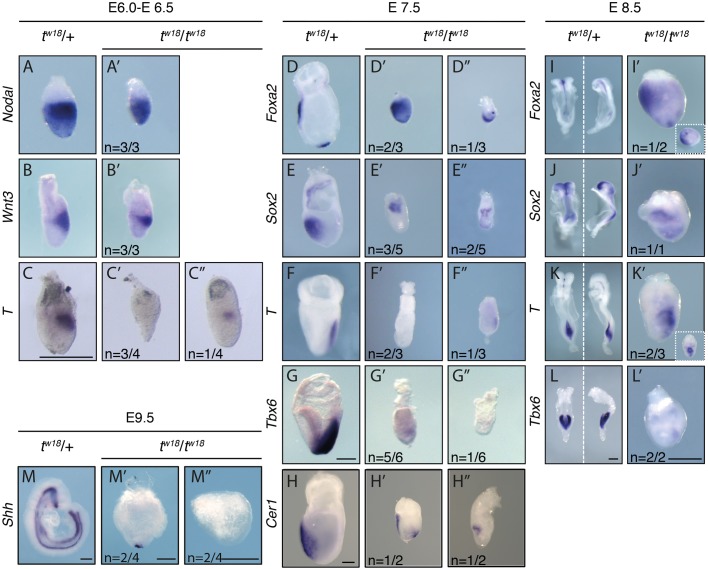
**Marker gene analyses by *in situ* hybridization highlight primitive streak and patterning defects in *t^w18^* mutant embryos.** (A-L″) WISH analysis of E6.5 (A-C″), E7.5 (D-H″), E8.5 (I-L′), and E9.5 (M-M″) *t^w18^* heterozygous (*t^w18^*/+) and homozygous (*t^w18^*/*t^w18^*) mouse embryos showing the expression of *Nodal* (A-A′), *Wnt3* (B-B′), *T* (C-C′, F-F″, J-J′), *Foxa2* (D-D″, H-H′), *Sox2* (E-E″, I-I′), *Tbx6* (G-G″, L-L′), *Cer1* (H-H″) and *Shh* (M-M″). Scale bars: 250 µM in A-G″ and I-M″, 200 µM in H-H″.

At E6.5, PS formation is initiated by activities of Nodal and Wnt3 signaling in the proximal posterior epiblast in concert with BMP signaling from the extraembryonic ectoderm (reviewed in Tam and Loebel, 2007). In all embryos derived from inter-crosses, *Nodal* and *Wnt3* mRNA showed the wild-type pattern according to the individual embryonic stage [mutant embryos were slightly younger (E6.0)] (Fig. 2A-B′). *T* expression in wild-type and heterozygous embryos overlapped with *Wnt3*, as expected (Fig. 2C). In contrast, *T* expression was undetectable (Fig. 2C′, *n*=3/4) or severely downregulated in *t^w18^* homozygotes (Fig. 2C″, *n*=1/4). Thus, the WISH data show that the signal molecules Nodal and Wnt3, which are essential for gastrulation and mesoderm formation, are expressed at their correct position in the *t^w18^* mutant. In contrast, activation of the downstream effector *T* is insufficient, indicating a deficiency in mesoderm formation.

From E7.5 onwards, homozygous *t^w18^* embryos can be identified morphologically by growth-retardation compared to wild-type and heterozygous littermates. At this stage, *Foxa2* marks the AVE and the node (Monaghan et al., 1993; Ang and Rossant, 1994; Perea-Gomez et al., 1999) (Fig. 2D). In homozygous *t^w18^* embryos *Foxa2* expression was detectable only in superficial cells probably representing the VE (Fig. 2D′,D″). In general, *Foxa2* transcripts appeared more widely expressed in homozygotes, with enrichment at either the distal tip or the presumed anterior pole. At E7.5, *Sox2* marks the future brain and the chorion (Wood and Episkopou, 1999; Avilion et al., 2003) (Fig. 2E). In *t^w18^* homozygotes, embryonic *Sox2* expression was either not detectable or reduced and without a clear anterior restriction (Fig. 2E′,E″). Extraembryonic ectodermal expression was detectable in all mutants but appeared as a band-like domain at the border to the epiblast (*n*=5). *T* expression was still either undetectable (Fig. 2F′, *n*=2/3) or faint (Fig. 2F″, *n*=1/3). *Tbx6*, a *T* target and marker of the paraxial mesoderm (Chapman et al., 1996, 2003) (Fig. 2G) was also only faintly expressed at the posterior end (Fig. 2G′, *n*=5/6) or undetectable (Fig. 2G″, *n*=1/6). In wild-type embryos *Cer1* was expressed in the AVE, as described (Fig. 2H) (Biben et al., 1998). Similarly, *Cer1* was detectable in the AVE of *t^w18^* homozygotes (Fig. 2H′,H″). In addition, in one of the mutants expression was detectable in migrating mesendoderm, as previously observed in early primitive streak-stage wild-type embryos (Fig. 2H′, *n*=1/2; see Fig. 4A in Biben et al., 1998).

Taken together, these data suggest that a functional AVE is present in the mutant embryos, but neuroectodermal patterning, as well as mesoderm and extraembryonic tissue development, is compromised in *t^w18^* homozygotes at E7.5.

E8.5 and older *t^w18^* mutants remain growth retarded, spherical and are disorganized. Organogenesis and axial elongation, as observed in wild-type and heterozygous littermates, is not taking place. Accordingly, the expression of marker genes lacks proper patterning. *Foxa2* was either undetectable (*n*=1/2, data not shown) or widely expressed in homozygotes (Fig. 2I′, *n*=1/2), and a small separate domain had formed at the distal tip of the embryo (Fig. 2I′ inlet), while heterozygous and wild-type littermates showed expression in the notochord and posterior endoderm. The pan-neural marker *Sox2* was diffusely and widespread expressed in *t^w18^* homozygotes (Fig. 2J′). The pan-mesodermal marker *T* was detectable in a patch of cells in some homozygotes (Fig. 2K′, *n*=2/3). *Tbx6* was hardly detectable in *t^w18^/t^w18^* embryos (Fig. 2L′, *n*=2/2).

At E9.5 *Shh* marks axial mesoderm and definitive endoderm (Martí et al., 1995) (Fig. 2M). In *t^w18^* mutants, a single domain of *Shh* expression at the distal tip of some embryos (Fig. 2M′, *n*=2/4) or no staining was observed (Fig. 2M″, *n*=2/4). The single distal domain of *Shh* expression might represent a node-like domain, given that a similar domain was observed for *Foxa2* expression at E8.5.

The marker expression patterns confirm that mesoderm formation (*T*), in particular paraxial mesoderm (*Tbx6*) and notochord development (*Foxa2, T*), remains impaired in E8.5 *t^w18^* homozygotes. Since the notochord and the mesoderm are essential for cellular organization and organ development, proper organ structures cannot form in the mutant.

**Fig. 3.**
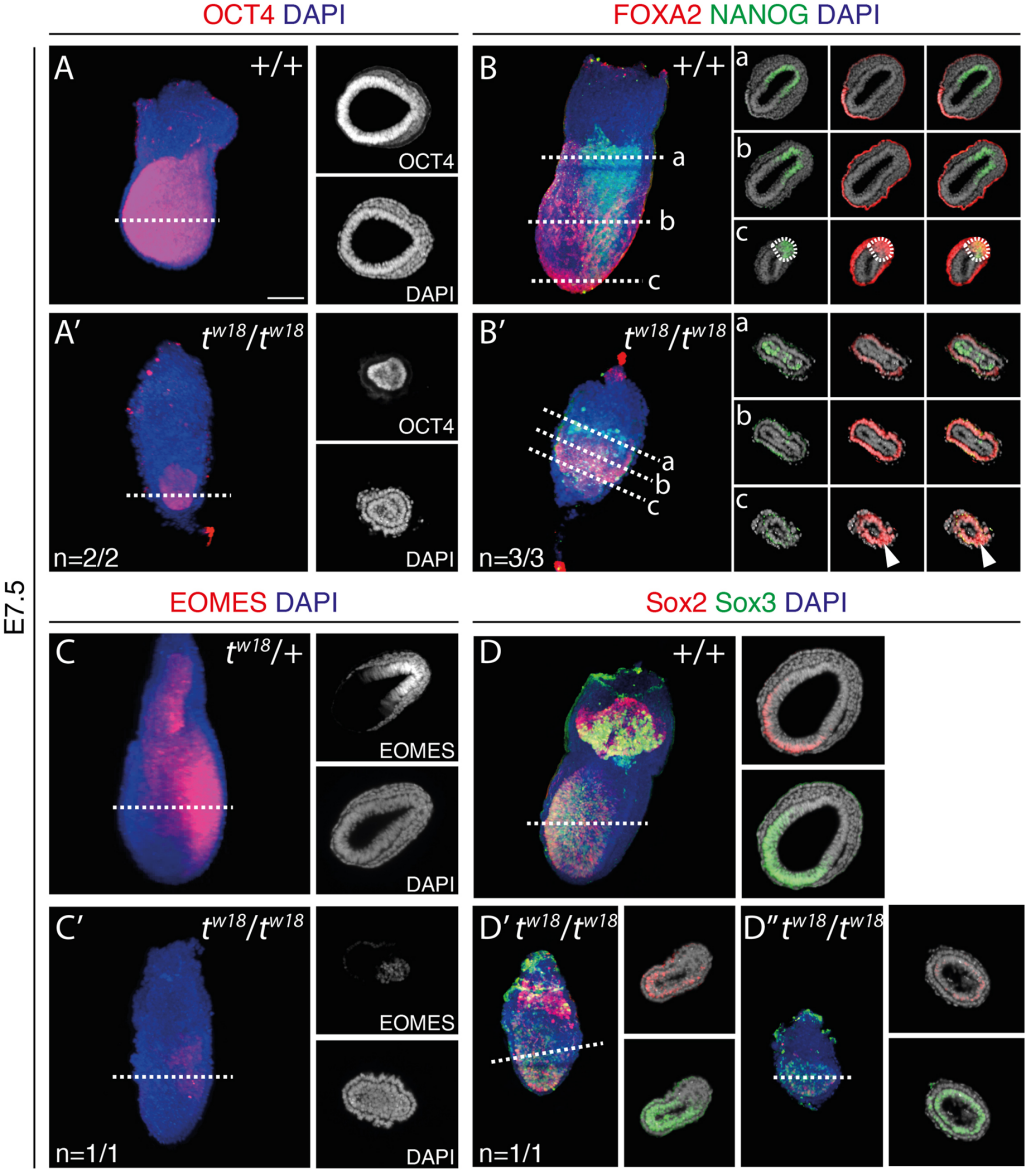
**Marker gene analyses by immunofluorescence staining highlight primitive streak and patterning defects in *t^w18^* mutant embryos.** (A-D″) E7.5 *t^w18^* heterozygous and homozygous mouse embryo whole-mount immunofluorescence staining for OCT4 (A-A′), FOXA2 and NANOG (B-B′), EOMES (C-C′), SOX2 and SOX3 (D-D″). Left big panels: lateral view of the embryo (3D volume renderings). Right small panels: optical transversal sections for the section planes indicated by white dashed lines in the left panels. Counterstaining was performed with DAPI. Scale bar: 100 µm.

To further characterize *t^w18^* mutants at the protein level, we performed whole-mount immunofluorescence staining for markers of pluripotency and differentiation on E7.5 embryos. The pluripotency factor POU5F1 (OCT4) is strongly expressed in the entire epiblast and quickly downregulated in differentiating cells of wild-type and *t^w18^* heterozygous embryos (Yeom et al., 1996; Yoshimizu et al., 1999; Hoffman et al., 2013) (Fig. 3A and data not shown). In *t^w18^* mutant embryos, the epiblast was small and malformed, but also OCT4 positive (Fig. 3A′).

At E7.5, FOXA2 protein marks the visceral endoderm, in particular the AVE, and the node; NANOG marks the posterior half of the epiblast, and EOMES the PS and nascent mesoderm (Fig. 3B,C). In homozygous *t^w18^* embryos, FOXA2 protein expression occurred in the right tissue (Fig. 3B′). An accumulation of strongly FOXA2-positive cells was detectable at the distal tip, in line with the node-like structure described before (Fig. 3B′, arrowhead in section plane c). NANOG protein expression was patchy and disorganized, restricted to the proximal epiblast, but without a posterior restriction (Fig. 3B′, section plane a). EOMES was weakly detectable in a patch of cells located at the presumed posterior end of a *t^w18^* mutant embryo, as part of a cell mass filling the amniotic cavity (Fig. 3C′).

With the onset of neurogenesis, the anterior epiblast expresses the neural control factors SOX2 and SOX3 (Wood and Episkopou, 1999) (Fig. 3C). Homozygous *t^w18^* embryos either expressed SOX2 and SOX3 with an anterior restriction (Fig. 3C′, *n*=1/2), or SOX2 protein was hardly detectable, while SOX3 was detected throughout the epiblast (Fig. 3C″, *n*=1/2). Accordingly, in *t^w18^* mutants neurogenesis appears to be initiated, while an anterior restriction does not occur in all cases.

**Fig. 4.**
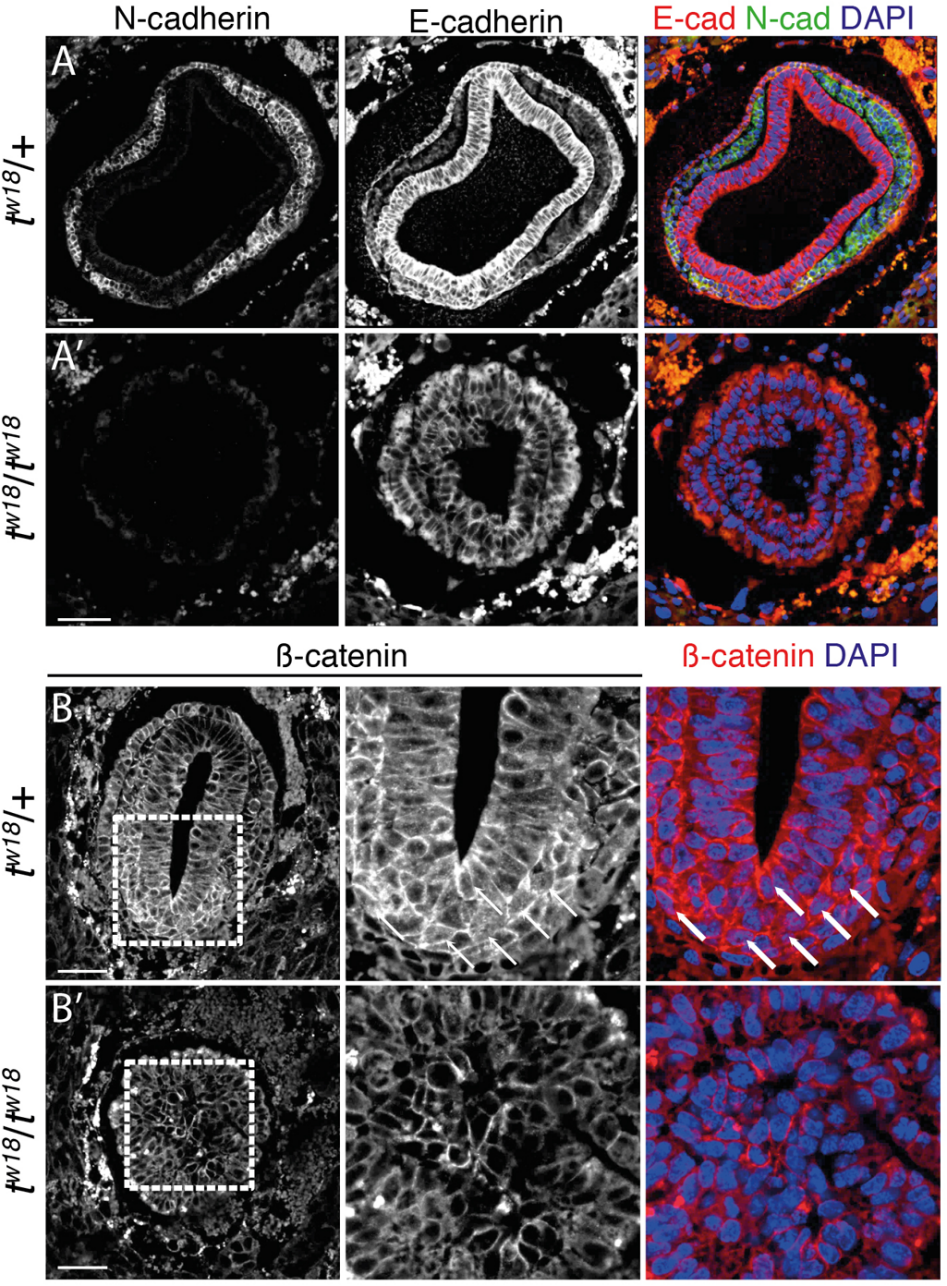
***t^w18^* mutant embryos are unable to undergo EMT.** (A-B′) Immunofluorescence staining on transversal paraffin sections of E7.5 *t^w18^* heterozygous (*t^w18^*/+) (A,B) and homozygous (*t^w18^*/*t^w18^*) (A′,B′) mouse embryos within the deciduum. Sections were probed for either E-Cadherin and N-Cadherin (A-A′) or β-Catenin (B,B′), and counterstained with DAPI. Dashed rectangles in B and B′ indicate regions magnified in the neighboring panels. Arrows in B point to some exemplary cells with nuclear β-Catenin staining. Scale bar: 50 µm.

Mesoderm formation involves epithelial to mesenchymal transition (EMT), a multistep process involving downregulation of E-cadherin (E-cad; *Cdh1*; Nakaya et al., 2008). In parallel, after uniform expression in the epiblast at E6.5, N-cadherin is up regulated (N-cad; *Cdh2*) in migrating mesodermal cells (Maeda et al., 2005; Wheelock et al., 2008; Bao et al., 2009) (Fig. 4A). In sections of *t^w18^* homozygous embryos, N-cad expression was not detectable, while E-cad protein was observed throughout the thickened epithelium (Fig. 4A′).

Another fundamental component of the adherens junctional complex is β-catenin (*Ctnnb1*; β-cat), which provides the intracellular link to the actin cytoskeleton, but also functions in the canonical WNT signaling pathway (Hulsken et al., 1994; reviewed in Kemler, 1993; Peifer, 1995). Accordingly, in sections of wild-type (not shown) and *t^w18^* heterozygous embryos, we observed β-cat at the membrane and also in nuclei at the PS (Fig. 4B). In sections of *t^w18^* homozygotes, β-cat was detected only at the membrane of epiblast cells and of cells filling the amniotic cavity (Fig. 4B′). These findings suggest that mutant cells retain epithelial characteristics, are unable to undergo EMT, and therefore accumulate at the site of the PS and bulge into the amniotic cavity.

The combined transcriptome, WISH, and immunofluorescence data provide a sufficiently detailed picture of the events leading to the *t^w18^* mutant phenotype. While the Nodal and Wnt3 signal molecules are properly expressed at the future posterior pole, the Nodal and WNT signal pathways in the epiblast are impaired and thus their targets are not or insufficiently expressed. The AVE is established in the mutants allowing neural induction at the anterior pole. PS induction by BMP signaling is likely to be initiated, and at E8.5 a node-like domain is present at the distal tip of some mutant embryos. However, notochord formation does not occur, mesoderm formation is impaired, and the embryo is disorganized. Since EMT does not occur either, proliferating cells in the posterior epiblast eventually fill the (pro)amniotic cavity in some of the embryos. However, some mesoderm formation downstream of BMP signaling appears to take place. There is also considerable variation in the penetrance of the mutant phenotype, which might be due to inhomogeneous (non-inbred) genetic backgrounds between embryos causing variable expression of the phenotype in individual embryos.

### The gastrulation defects of homozygous *t^w18^* mutants are caused by loss of *Ppp2r1a*

**Fig. 5.**
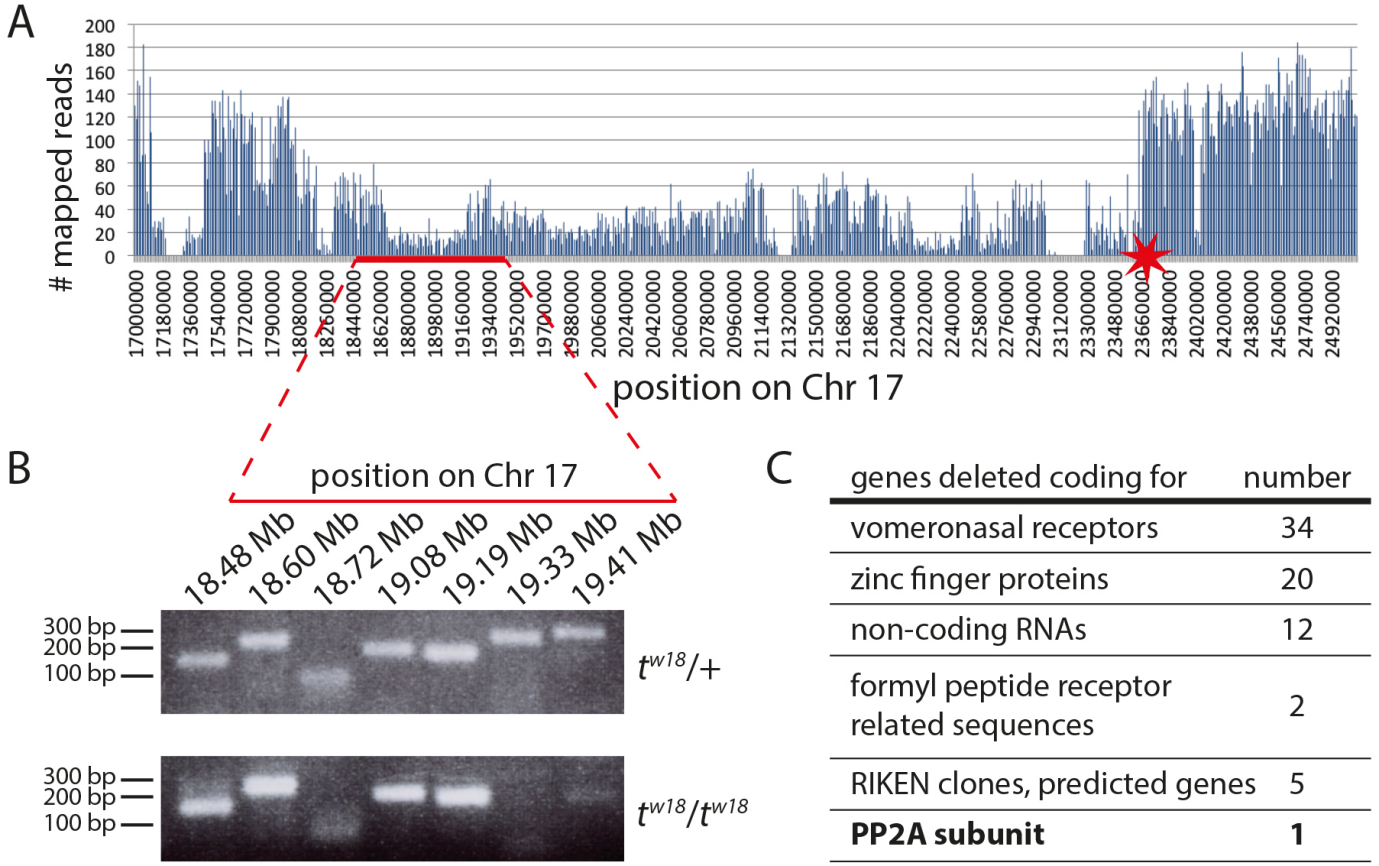
**The *t^w18^* deletion extends over 4.3 Mbp of Chromosome 17.** (A) Number of reads obtained by next generation sequencing of *t^w18^*/+ derived genomic DNA mapped to position Chr 17:17000000-25090000. The 3′ end of the deletion is found approximately at position Chr 17:23600000 (asterisk) whereas the 5′ end was roughly positioned between Chr 17: 18.4-19.4 Mb. (B) The presence of DNA elements around the region determined to contain the proximal breakpoint were analyzed by PCR. DNA fragments obtained by PCR amplification and visualized by gel electrophoresis; homozygous *t^w18^* genomic DNA (lower panel) was amplified up to position Chr 17:19.19 Mb, whereas amplicons were absent from position Chr 17:19.33 Mb, indicating that the deletion starts between position 19.19 and 19.33 Mb. (C) Rough functional assignment of the 74 genes deleted in *t^w18^*.

Next we attempted to identify the gene responsible for the *t^w18^* mutant phenotype. The *t^w18^* haplotype is characterized by a large deletion of at least 3.3 Mb of the *t* complex on Chr17 (Búcan et al., 1987; Barclay et al., 1996). Since all protein-coding genes have been identified by genome sequencing, we decided to take a candidate gene approach to identify the *t^w18^* lethal. This required us to determine the breakpoints and exact extent of the deletion, and thus all genes deleted. We attempted to generate *t^w18^* homozygous embryonic stem cell (ESC) lines, which failed. Therefore we sequenced the genomic DNA of heterozygous *t^w18^*/+ by massive parallel sequencing. Read mapping to Chr17 revealed a sharp increase of the read density at position 23.6 Mb marking the distal deletion breakpoint (Fig. 5A, asterisk). On the centromere-close side only an approximate interval of the deletion breakpoint could be demarcated between Chr 17:18.0-19.7 Mb (Fig. 5A, red line). To position the breakpoint more precisely, we performed polymerase chain reaction (PCR) analyses on genomic DNA derived from heterozygous *t^w18^*/+ and homozygous *t^w18^/t^w18^* embryos. Amplicons could be generated until position Chr17:19190492 in both DNA samples (Fig. 5B, 18.48-19.19 Mb), whereas fragments from Chr17:19330513 on could not be amplified from *t^w18^/t^w18^* genomic DNA (Fig. 5B, 19.33 and 19.41 Mb, lower panel). Thus, we defined the region between Chr17:19190492-19330513 and 23.6 Mb, an interval of 4.3 Mb, as the deletion of the *t^w18^* haplotype carrying the *t^w18^* lethal.

The entire deletion contains 74 genes (Fig. 5C; Table S2). We excluded the 34 vomeronasal receptor genes and the non-coding RNAs as likely candidates, and searched the databases and literature for information on the remaining 25 candidates. For most of the 20 zinc finger genes the available data provided no information on expression or function.

**Fig. 6.**
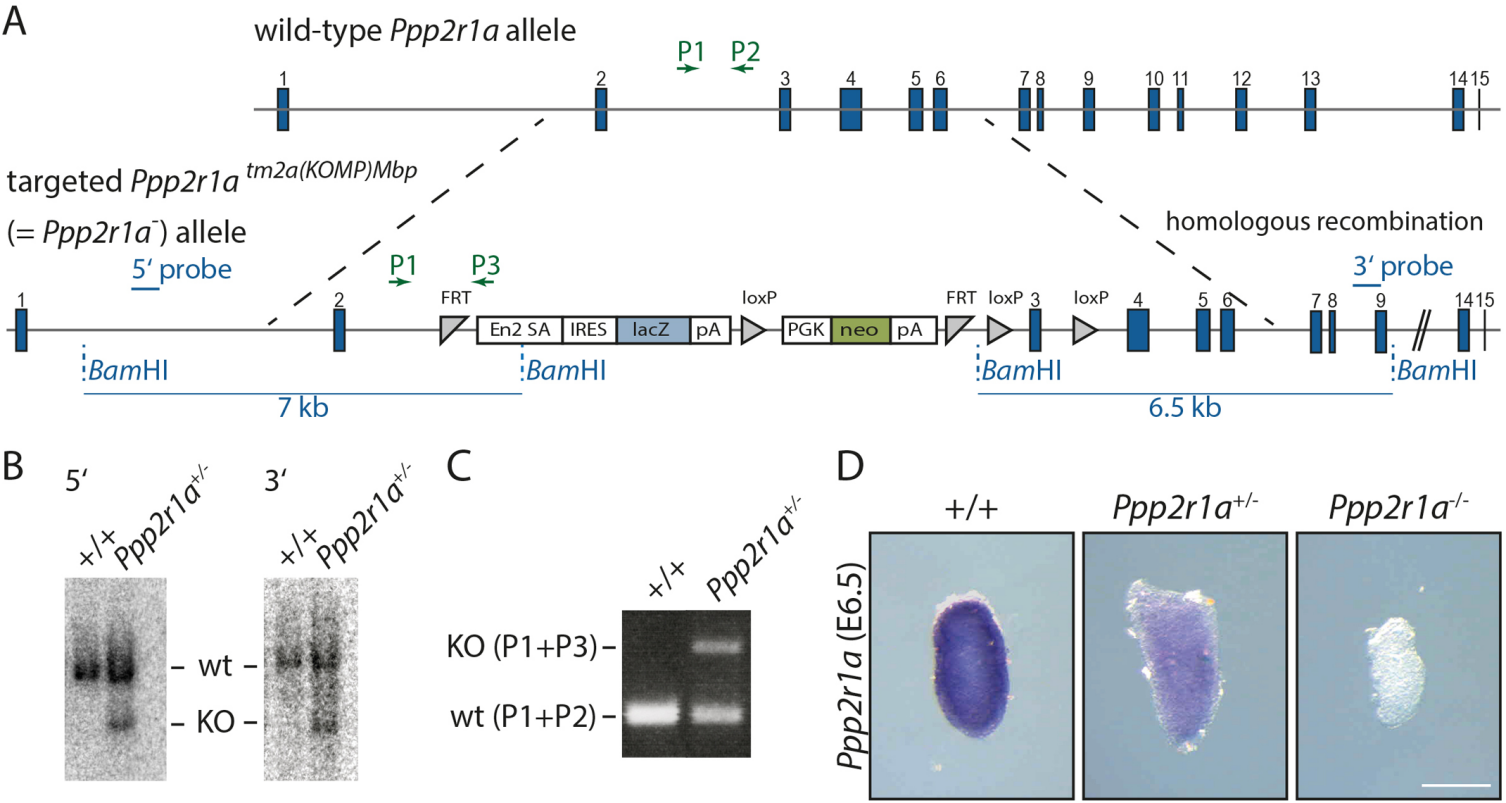
**Targeting of the *Ppp2r1a* locus by homologous recombination.** (A) *Ppp2r1a* targeting construct integrated into the wild-type *Ppp2r1a* locus (upper panel) by homologous recombination (lower panel). The stop cassette in intron 2 leads to abrogation of transcription. (B) Southern blot analysis of a successfully targeted ESC clone; a *Bam*HI digest reveals a wild-type fragment of 13 kb, the targeted allele shows a 7 kb fragment with the 5′ and a 6.5 kb fragment with the 3′ external Southern probe. (C) PCR genotyping using three primers (P1, P2, P3) distinguishes between wild-type mice and mice carrying the targeted allele. (D) WISH analysis of embryos at E6.5 resulting from *Ppp2r1a*^+/−^ inter-crosses shows absence of *Ppp2r1a* expression in homozygous *Ppp2r1a*^−/−^ embryos. Scale bar: 200 µm.

The gene encoding the PP2A scaffolding subunit Aα, *Ppp2r1a* (Mayer-Jaekel and Hemmings, 1994) was selected as the most likely candidate, since PP2A plays an important role in signal transduction. In addition, homozygous loss of the catalytic subunit Cα (*Ppp2ca*) of PP2A has been shown to cause mesodermal defects and embryonic lethality (Götz et al., 1998). However, PPP2R1A can be substituted in the PP2A complex by PPP2R1B, and it was uncertain if loss of *Ppp2r1a* would be complemented by *Ppp2r1b* in the gastrulating embryo. To test this we generated a *Ppp2r1a* knockout in G4 ESCs (George et al., 2007) using a construct provided by the KOMP consortium (Fig. 6A). We screened for homologous recombination by using Southern blot analysis (Fig. 6B). We generated heterozygous mice and established a multiplex PCR approach to facilitate genotyping of mutants (Fig. 6C).

To determine the expression pattern of *Ppp2r1a* in embryos and to validate the loss of function mutation, we performed WISH of *Ppp2r1a* RNA on E6.5 embryos obtained from *Ppp2r1a*^+/−^ inter-crosses. *Ppp2r1a* mRNA is ubiquitously expressed in wild-type and heterozygous embryos (Fig. 6D). Homozygous littermates lacked the *Ppp2r1a* transcript, demonstrating that the knockout allele is a true null allele.

**Fig. 7.**
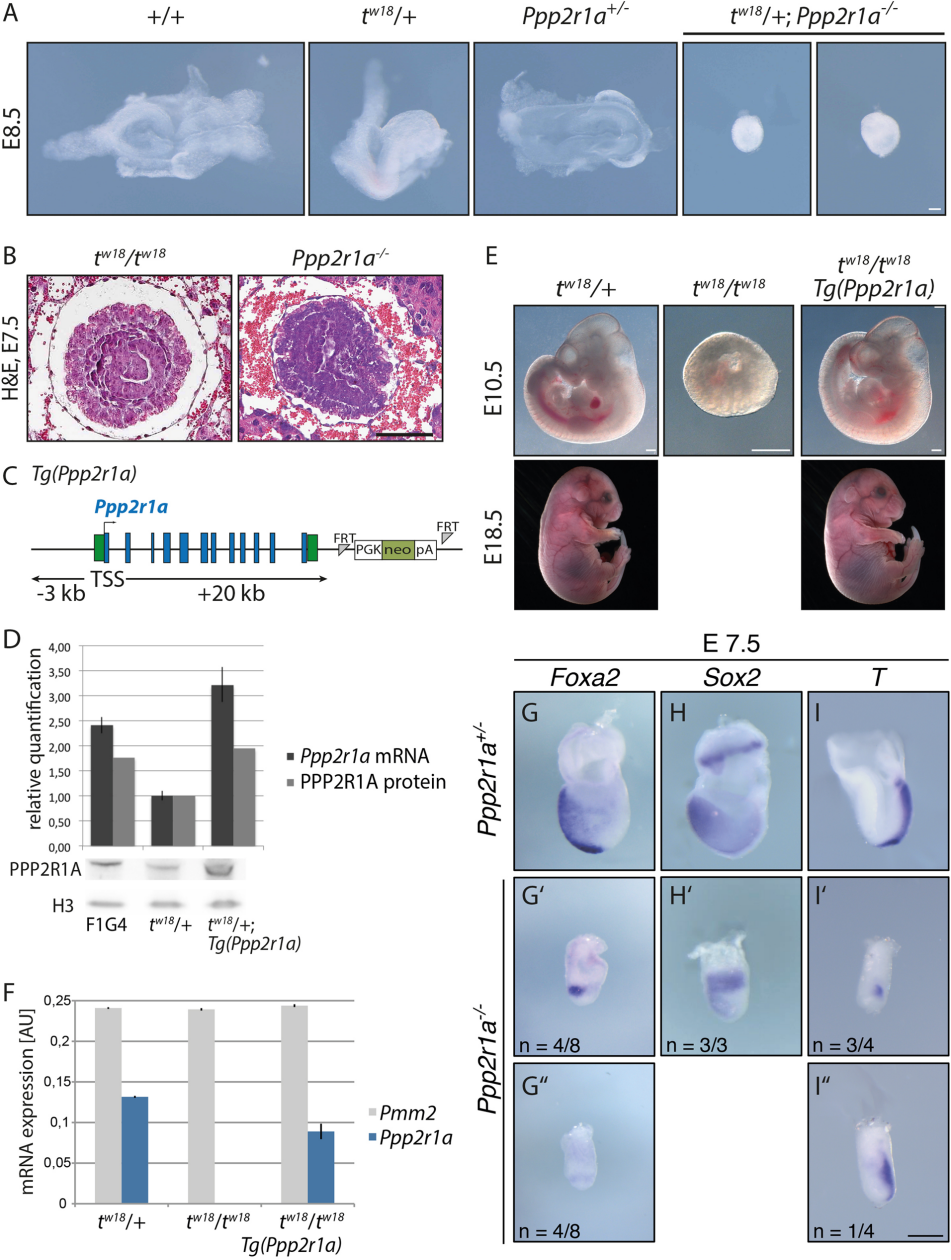
**Knockout of *Ppp2r1a* reconstitutes and *Ppp2r1a* expression rescues the *t^w18^* homozygous phenotype.** (A) Morphology of E8.5 embryos derived by crossing *t^w18^*/+ and *Ppp2r1a*^+/−^ mice; embryos were genotyped by PCR following imaging. *t^w18^*/+;*Ppp2r1a*^−/−^ embryos failed to gastrulate and resemble *t*^w18^ homozygotes. (B) Histological H&E-stained paraffin sections of E7.5 *Ppp2r1a* knockout and *t^w18^*/*t^w18^* embryos. The amniotic cavities of both embryos are filled with cells. (C) Rescue construct containing the *Ppp2r1a* locus comprising 3 kb upstream and 20 kb downstream of the transcription start site (TSS). (D) Test for functioning of the *Ppp2r1a* rescue construct in ESCs. *Ppp2r1a* RNA expression analyzed by qPCR and normalized to *Pmm2* and *Hmbs* (upper panel), PPP2R1A protein levels analyzed by western blotting (lower panel) followed by quantification (upper panel) relative to H3 protein. Expression of the rescue transgene increased *Ppp2r1a* mRNA and protein levels in the transgenic ESC clone. Error bars indicate standard deviation of technical replicates. (E) *Ppp2r1a* expression rescues the *t^w18^* homozygous phenotype. Embryos from breedings of *t^w18^*/+ females with *t^w18^*/+, Tg(*Ppp2r1a*) males were analyzed at E10.5 and E18.5; homozygous *t^w18^* embryos carrying the transgene Tg(*Ppp2r1a*) developed normally at least until E18.5. (F) qPCR analyses of E10.5 embryos from (E). *Ppp2r1a* expression levels were restored to 67% of the single wild-type allele of *t^w18^* heterozygotes by the rescue construct. Error bars indicate standard deviation of technical replicates. (G-I″) WISH analysis of E7.5 *Ppp2r1a^+/−^* and *Ppp2r1a^−/−^* mouse embryos for the expression of *Foxa2*, *Sox2*, and *T*. Scale bar: 100 µm in A,G-I″ and 500 µm in E.

Next we tested if the *Ppp2r1a* knockout allele would complement the *t^w18^* phenotype or cause a phenotype in embryos carrying both mutant alleles (Fig. 7A). *Ppp2r1a*^+/−^ mice were mated with *t^w18^*/+ mice and embryos were dissected at E8.5. Embryos carrying *t^w18^* and the *Ppp2r1a* knockout allele on the wild-type chromosome (*t^w18^*/+; *Ppp2r1a^−/−^*) showed growth retardation and no organ structures, while heterozygous littermates of either genotype had developed normally. These data showed that the *Ppp2r1a* knockout uncovers a developmental lethal within the *t^w18^* deletion and does not complement the *t^w18^* lethal phenotype. Next we asked if the mutant phenotype of the *Ppp2r1a* knockout and of *t^w18^* homozygous embryos is similar (Fig. 7B). Histological sections of homozygous embryos showed that the amniotic cavity was filled with cells in both mutants at E7.5. This similarity of the phenotype suggested that the *t^w18^* lethal phenotype indeed may be due to loss of *Ppp2r1a.*

The morphological data, however, did not exclude that the *t^w18^* deletion might contain another gene showing a similar phenotype upon loss of function, and that the *t^w18^* homozygous phenotype may be the result of several gene losses. To exclude the latter possibility we performed a rescue experiment. We subcloned the *Ppp2r1a* gene including 3 kb upstream and 20 kb downstream of the TSS comprising the gene promoter and poly(A) signal via gap repair recombineering from a bacterial artificial chromosome (BAC) clone and introduced the linearized clone as a transgene randomly into *t^w18^*/+ ESCs (Fig. 7C). We analyzed *Ppp2r1a* mRNA levels by using quantitative PCR (qPCR) and analyzed protein levels by using western blotting in wild-type (F1G4), *t^w18^*/+ and transgenic ESCs (Fig. 7D). As expected, *Ppp2r1a* expression was around twofold higher in the wild-type cells compared to *t^w18^*/+ cells, which express only one allele. The transgenic construct increased the mRNA and protein expression of *Ppp2r1a* in transgenic ESCs to approximately the wild-type level, suggesting that it functions properly (Fig. 7D).

We established a mouse line from transgenic ESCs by morula aggregation (Eakin and Hadjantonakis, 2006). We mated transgenic mice with *t^w18^* heterozygotes and isolated embryos at E10.5. The *t^w18^* homozygous embryos, which had survived to this stage, did not show recognizable organ structures, while homozygotes carrying the transgene [*t^w18^*/*t^w18^*; Tg (*Ppp2r1a*)] were morphologically indistinguishable from heterozygotes (Fig. 7E). The rescue was also complete at E18.5 suggesting that the *t^w18^* deletion contains no other genes besides *Ppp2r1a*, which are essential for embryogenesis (Fig. 7E). qPCR data confirmed the expression of *Ppp2r1a* mRNA in transgenic embryos homozygous for *t^w18^* (Fig. 7F).

Thus, loss of *Ppp2r1a* is solely responsible for the embryonic lethal phenotype caused by the *t^w18^* deletion.

To further confirm that the molecular phenotype of *Ppp2r1a*^−/−^ embryos showed the same expression patterns of marker genes as *t^w18^* homozygotes, we performed WISH on E7.5 embryos. The expression of *Foxa2*, *Sox2*, and *T* showed very similar dysregulation in *Ppp2r1a*^−/−^ as in *t^w18^* homozygotes at this stage, though the severity of the malformation was variable probably due to mixed genetic background of the mutants (compare Fig. 7G-I″ to Fig. 2D-F″).

In summary, we were able to demonstrate beyond doubt that the *t^w18^* lethal is due to loss of *Ppp2r1a*.

## DISCUSSION

The *Brachyury* (*T*) mutation was the first ‘classical’ lethal mutation identified, which affects mesoderm formation and axial development (Chesley and Dunn, 1936). Studying its properties led to the discovery of the mouse *t* haplotype and, over decades, to the identification of 16 recessive lethal mutations, the ‘*t* lethals’, causing early embryonic death (Ruvinsky et al., 1991; Bennett, 1975). Therefore, some four decades ago, the *t* complex (the wild-type form of the *t* haplotype) has been speculated to represent the prime genomic region encoding control genes for early embryonic development in the mouse. However, this proposition did not hold true. Attempts made in the following decades to isolate *t* lethals have been hampered by the difficulties to precisely localize the responsible genes within the *t* haplotype, and despite recent technical advances, prior to this report only a single *t* lethal, *t^w5^*, had been identified and molecularly characterized (Sugimoto et al., 2012).

Here we have shown that the *t* lethal *t^w18^* is a loss-of-function mutation of the gene encoding the PP2A scaffold protein PPP2R1A, which is located within a large deletion of approximately 4.3 Mbp in the *t^w18^* haplotype. Via genetic rescue of the *t^w18^* deletion by a *Ppp2r1a* transgene we show that none of the other 73 genes including 20 zinc finger genes located in this deletion is essential for embryonic development. We provide a molecular characterization of the dysfunction leading to the gastrulation defects described for homozygous embryos of the *t^9^* complementation group (*t^4^, t^9^, t^w18^*) about five decades ago (reviewed in Bennett, 1975).

The WNT and Nodal signaling pathways play essential roles in embryonic pattern formation, gastrulation and differentiation. *Nodal* and *Wnt3* expression at the early streak stage (E6.0-E6.5) appeared largely unaffected in homozygous *t^w18^* embryos, whereas their target genes were downregulated (Fig. 5D), suggesting defects in signal transduction of these pathways. Therefore, *Ppp2r1a* functions at some components of these signaling cascades.

PP2A activity is mediated by assembly of the core enzyme, consisting of the structural A subunit (PPP2R1A or PPP2R1B) and the catalytic C subunit (PPP2CA, PPP2CB), with a variable regulatory B subunit mediating substrate specificity (Sents et al., 2013). There are several lines of evidence for a role of PP2A in WNT signaling, both as a negative and a positive regulator of the cascade (reviewed in Eichhorn et al., 2009; Seeling et al., 1999; Li et al., 2001; Yamamoto et al., 2001). The downregulation of WNT targets in *t^w18^* mutants provides good evidence that in the context of gastrulation of mouse embryos PP2A is required as an activator in the WNT signaling pathway.

Bajpai et al. (2004) showed that in *Drosophila*, loss of the homologue of a B subunit of PP2A, PR55, results in downregulation of WNT targets, suggesting a role of PP2A:PR55α in stabilizing cytoplasmic β-catenin. This role is supported by the finding that PP2A is required for β-catenin dephosphorylation thereby inhibiting its degradation *in vivo* (Zhang et al., 2009; Park et al., 2016). Next to the B regulatory subunits, also the scaffolding subunit itself might contribute to the regulation of the canonical WNT pathway. In malignant colon cancer cell lines, which display a highly activated WNT pathway, PPP2R1A is almost exclusively used to assemble the PP2A holoenzyme, while in normal colon cell lines, both the α and β scaffolds are present (Carmen Figueroa-Aldariz et al., 2015). Thus, it appears that PPP2R1A is preferred over PPP2R1B in regulating WNT signaling. In *t^w18^* homozygous embryos, the lack of PPP2R1A might be partially complemented by the β scaffolding subunit, providing a possible explanation for the variable severity of the mutant phenotype.

The second developmentally important signaling cascade found to be dysregulated in *t^w18^* homozygous embryos is the Nodal pathway. It was shown that the PR55α B regulatory subunit (PPP2R2A) can interact with type I TGFβ receptors (Griswold-Prenner et al., 1998). A later study showed that PR55α (PPP2R2A) and the closely related B regulatory subunit PR55δ (PPP2R2D) exert opposing effects on the TGFβ/Activin/Nodal pathway by regulating type I receptor stability and activity (Batut et al., 2008). Knockdown of PR55α or overexpression of PR55δ in *Xenopus* embryos results in anteroposterior axis truncation, resembling phenotypes of mutants with reduced Nodal signaling. Indeed, expression levels of the Activin/Nodal target genes, such as *Gsc* were strongly reduced in these embryos. However, the exact molecular mechanisms behind these effects have so far not been elucidated. Since a reduced expression of Nodal targets was observed in mouse embryos lacking *Ppp2r1a*, it is possible that PPP2R1A in complex with PR55α is required for positive regulation of Nodal signaling by stabilizing type I TGFβ receptors, as has been shown in *Xenopus*. Another line of evidence comes from the knockout phenotype of the Activin receptor type IIa in combination with a heterozygous type IIb or a heterozygous Nodal mutation, which have been shown to cause growth arrest at the egg cylinder stage and to affect PS and mesoderm formation, reminiscent of the *t^w18^* lethal phenotype (Song et al., 1999). Thus, the growth retardation observed in *t^w18^* homozygous embryos might relate to impaired Nodal signaling in the mutant epiblast. The combined data provide compelling evidence for an essential role of *Ppp2r1a* in Nodal signal transduction during gastrulation of the mouse embryo. The spatiotemporal expression of the different PP2A subunits during early embryogenesis and how the type I receptor activity is affected in *t^w18^* embryos requires further investigation.

Besides dysregulation of the WNT and Nodal signaling cascades, the gene expression profile of E6.5 homozygous *t^w18^* embryos showed an upregulation of genes involved in cell adhesion. Mutant cells move towards the streak but then fail to delaminate and become stalled in that region. Under wild-type conditions, cells ingressing through the PS undergo EMT, which includes a switch from *E-cad* to *N-cad* expression that provides cells with a migratory behavior (Maeda et al., 2005; Wheelock et al., 2008). In our transcriptome data *Eomes* expression was downregulated in *t^w18^* mutants. Arnold et al. (2008) showed that mutants lacking *Eomes* specifically in the epiblast display defects in EMT and maintenance of *E-cad* expression. We identified a similar persistence of E-cad, accompanied by a lack of N-cad in the embryo proper of E7.5 *t^w18^* homozygotes (Fig. 4A-B″). Thus, the defects in EMT observed in *t^w18^* mutant embryos might be secondary to the downregulation of *Eomes*.

The exact molecular mechanisms by which PPP2R1A contributes to the regulation of the WNT and Nodal signaling pathways, and in particular the protein targets of PP2A in these signaling pathways, remain to be investigated.

## MATERIAL AND METHODS

### Mice and embryos

Mice were maintained in the animal facility of the Max Planck Institute for Molecular Genetics, Berlin, in accordance with international standards and protocols. Animal maintenance and all procedures performed on mice described here were performed in accordance with the German Animal Welfare Act (Tierschutzgesetz) and had prior approval from local authorities (LAGeSo).

### Generation of ESC lines and transgenic mouse lines

We generated a *t^w18^*/+ embryonic stem cell line according to standard procedures (Behringer et al., 2014). Blastocysts were obtained from matings of *t^w18^*/+ males kept on a 129/SvEv genetic background to *C57BL/6* females. To knock out *Ppp2r1a* G4 ESCs (George et al., 2007) were targeted by homologous recombination using a construct (PRPGS00108_A_B03) obtained from the KOMP repository. Clones were screened for successful homologous recombination by Southern blot analysis using external 5′ and 3′ probes. For the rescue experiment the transgene construct *Tg(Ppp2r1a)* containing only the *Ppp2r1a* locus was generated from a BAC (RPCI-23-209P21, Osoegawa et al., 2000) by Red/ET recombineering (Muyrers et al., 1999) and randomly integrated in *t^w18^*/+ ESCs. Successful integration was verified by PCR (see genotyping). Mouse lines were generated from transgenic ESCs by morula aggregation (Eakin and Hadjantonakis, 2006).

### Genotyping

Mouse ear biopsies, embryos and sections were genotyped by PCR under standard conditions using the following primer pairs: *t^w18^*: Vil2s 5′-tcatggaccaacacaagctc-3′, Vil2as 5′-cacaaaactgaaatctccctc-3′(wt: 180 bp, *t^w18^*: 230 bp); *Ppp2r1a* KO alleles: fwd1 5′-gcaggagctggagcatgcca-3′, rev1 5′-tgcagcacaggatgttcagtgga-3′, rev2 5′-ggagagggacctggctcctatg-3′ (wt: 258 bp. KO: 436 bp); *Tg(Ppp2r1a)*: fwd 5′-ttttgctgctgtaatggggacc-3′, rev 5′-gctaaagcgcatgctccaga-3′ (293 bp).

### WISH

For WISH including synthesis of DIG-labeled antisense probes the protocol provided by the MAMEP database (http://mamep.molgen.mpg.de) was used. The probe for *Ppp2r1a* corresponds to nucleotides 930-1858 of NM_016891.3. Probe templates were amplified using standard PCR procedure with a reverse primer containing a T7 site for antisense transcription. *In situ* hybridizations for *Foxa2* (clone name: UP001), *T* (clone name: 9226), *Tbx6* (clone name: 9310), *Sox2* (clone name: UL027), and *Cer1* (clone name: CE232) were performed using probes from MAMEP. The *Nodal* probe template was kindly provided by the Robertson laboratory and linearized using *Bam*HI. The *Wnt3* probe template was kindly provided by the McMahon laboratory.

### Histology

Whole deciduae were fixed in 4% paraformaldehyde (PFA)/phosphate-buffered saline (PBS) overnight, processed into paraffin and sectioned at 5 µm. Hematoxylin and eosin (H&E) staining was performed according to standard procedures. For immunofluorescence staining, sections were de-paraffinized using xylene and rehydrated in an ethanol serious. For antigen retrieval, sections were boiled in 0.01 M citrate buffer pH 6.0 for 10 min. Blocking was performed with 2% bovine serum albumin/2% lamb serum/PBS. The following antibodies were diluted in blocking solution and incubated overnight at 4°C: anti-E-cadherin (610181, BD Biosciences, 1/200), anti-N-cadherin (13116, CST, 1/100), and anti-β-catenin (610153, BD Biosciences, 1/200). Sections were incubated with the secondary antibodies for 1 h at room temperature (RT): anti-rabbit IgG-Alexa488 conjugated (A-11034, Thermo Fisher Scientific, 1/500) and anti-mouse IgG-Alexa546 conjugated (A-11030, Thermo Fisher Scientific, 1/500). Sections were mounted with VECTASHIELD HardSet Mounting Medium with DAPI (Vector Laboratories).

### Whole-mount immunofluorescence staining and clearing

For whole-mount immunofluorescence, E7.5 embryos were dissected in ice-cold PBS and processed according to the protocol provided by Abcam (www.abcam.com). Primary antibodies were applied for 48-72 h in blocking solution: anti-OCT4 (sc-8628, Santa-Cruz Biotechnology, 1:200), anti-Sox2 (AF2018, R&D systems, 1:200), anti-Sox3 (provided by L. Gunhaga, Umeå Centre for Molecular Medicine, Umeå University, Sweden, 1:200) anti-Nanog (ab80892, Abcam, 1/100) anti-FoxA2 (sc-6554, Santa Cruz Biotechnology, 1/200), and anti-Eomes (ab23345, Abcam, 1/200). Secondary antibodies were applied in blocking solution overnight: anti-rabbit IgG-Alexa488 conjugated (A-21206, Thermo Fisher Scientific, 1/200) and anti-goat IgG-Alexa594 conjugated (A-11058, Thermo Fisher Scientific, 1/500). For clearing, specimens were postfixed in PBS/4% PFA for 20 min at 4°C, rinsed twice in 0.02 M phosphate buffer (PB) pH 7.4 and incubated in refraction index matching medium (RIMS, Yang et al., 2014) until transparency was reached. Before lightsheet imaging, the samples were embedded in PBS/1.5% low-melting point agarose and incubated in RIMS for another 24 h.

### Deletion mapping by next-generation DNA sequencing and PCR

Genomic DNA was extracted from wild-type and *t^w18^*/+ ESCs. Sequencing was performed using the Illumina HiSeq 2000 system (shotgun sequencing) for 36 bp single reads that were mapped to the mouse genome mm10. PCR according to standard procedures was performed using the following primers: 18.5 Mb fwd 5′-agtccgaggccacgtccttga-3′, rev 5′-gctgcagttgctgtgctggc-3′; 18.6 Mb fwd 5′-ggccttccattcggtcctggg-3′, rev 5′-aggagtgctgtgctggacgc-3′; 18.7 Mb fwd 5′-tcttccccagaggacccggc-3′, rev 5′-gcagctgcagtggtgggcag-3′; 19.1 Mb fwd 5′-gccaacctgagaacgcgctg-3′, rev 5′-agggaaatgcctggggcagga-3′; 19.2 Mb fwd 5′-gccggccagcagctcacata-3′, rev 5′-ggcagcgtgccccactgaat-3′; 19.3 Mb fwd 5′-ccccggcatatcctgcgtagatga-3′, rev 5′-gggtgatgggcgagcaacaaatca-3′; 19.4 Mb fwd 5′-agccccatgcccagcgacta-3′, rev 5′-agtggctgtctggcccacca-3′.

### RNA extraction, qRT-PCR and RNA-seq

RNA was extracted from cells or embryo tissues using the RNeasy Mini or Micro Kits (Qiagen). cDNA was synthesized from total RNA using the QuantiTect^®^ Reverse Transcription Kit (Qiagen). qPCR was performed using the GoTaq^®^ qPCR Master Mix (Promega) on the StepOnePlus Real-Time PCR System (Life Technologies). The following primer pairs were used: *Ppp2r1a*: fwd 5′-ccaccaagcacatgctgccc-3′, rev 5′-tccacatcctggtcctgggtca-3′; *Pmm2*: fwd 5′-agggaaaggcctcacgttct-3′; rev 5′-aataccgcttatcccatccttca-3′.

For RNA-seq the TotalScript™ RNA-Seq Kit (epicenter) was used for cDNA synthesis and library preparation. The sequencing was performed using the Illumina HiSeq 2500 (High Output) system for 50 bp paired end reads. Reads were mapped to the mouse genome mm10 (GRCm38) with TopHat (Trapnell et al., 2009) (v2.0.8b) and Cuffdiff (Trapnell et al., 2010) was used to calculate normalized FPKM values. Heat maps of differentially expressed genes and PCC were generated using the R program (http://www.r-project.org).

### Immunoblotting

Whole-cell lysates were prepared using Western lysis buffer (1% SDS, 2 mM ethylenediaminetetraacetic acid, 10 mM Tris; pH 8.0). Immunoblotting was performed following standard protocols with anti-Ppp2r1a (6F9, Abcam, 1:1000) and anti-H3 (ab1791, Abcam, 1:10,000) antibodies. Horseradish peroxidase-linked secondary antibodies were obtained from Cell Signaling Technologies. Signals were imaged and quantified using the Fusion SL Vilber Lourmat system (Peqlab).

### Imaging

For imaging of WISH-stained embryos, a MZ16A dissection microscope (Leica) fitted with an AxioCam MRc5 (Carl Zeiss MicroImaging) in combination with AxioVision software (Carl Zeiss MicroImaging), and a Zeiss Axio Zoom.V16 in combination with an Axiocam 512 color and ZEN software (Carl Zeiss MicroImaging), were used. H&E-stained paraffin sections were imaged using a Zeiss Observer.Z1 microscope fitted with an AxioCam MRc (Carl Zeiss MicroImaging) and AxioVision software. Immunofluorescence stainings on paraffin sections were imaged using an LSM 710NLO (Carl Zeiss MicroImaging) and ZEN software (Carl Zeiss MicroImaging). Immunofluorescent embryos were imaged in RIMS (Yang et al., 2014) using a Lightsheet Z.1 (Carl Zeiss MicroImaging) and ZEN software.
